# Taking One’s Own Life in Hospital? Patients and Health Care Professionals Vis-à-Vis the Tension between Assisted Suicide and Suicide Prevention in Switzerland

**DOI:** 10.3390/ijerph15061272

**Published:** 2018-06-15

**Authors:** Stella Reiter-Theil, Charlotte Wetterauer, Irena Anna Frei

**Affiliations:** 1 Department Clinical Ethics, Psychiatric Hospitals of the University Basel (UPK), University Hospital Basel (USB), University Basel, 4002 Basel, Switzerland; charlotte.wetterauer@usb.ch; 2 Department Nursing and Allied Health Professions, University Hospital Basel, 4031 Basel, Switzerland; IrenaAnna.Frei@usb.ch

**Keywords:** suicide, prevention, assisted suicide, Swiss right-to-die societies, ethics support, ethics policy, law, health professional

## Abstract

In Switzerland, the practice of lay right-to-die societies (RTDS) organizing assisted suicide (AS) is tolerated by the state. Patient counseling and accompaniment into the dying process is overtaken by RTDS lay members, while the role of physicians may be restricted to prescribing the mortal dose after a more or less rigorous exploration of the patient’s decisional capacity. However, Swiss health care facilities and professionals are committed to providing suicide prevention. Despite the liberal attitude in society, the legitimacy of organized AS is ethically questioned. How can health professionals be supported in their moral uncertainty when confronted with patient wishes for suicide? As an approach towards reaching this objective, two ethics policies were developed at the Basel University Hospital to offer orientation in addressing twofold and divergent duties: handling requests for AS and caring for patients with suicidal thoughts or after a suicide attempt. According to the Swiss tradition of “consultation” (“Vernehmlassung”), controversial views were acknowledged in the interdisciplinary policy development processes. Both institutional policies mirror the clash of values and suggest consistent ways to meet the challenges: respect and tolerance regarding a patient’s wish for AS on the one hand, and the determination to offer help and prevent harm by practicing suicide prevention on the other. Given the legal framework lacking specific norms for the practice of RTDS, orientation is sought in ethical guidelines. The comparison between the previous and newly revised guideline of the Swiss Academy of Medical Sciences reveals, in regard to AS, a shift from the medical criterion, end of life is near, to a patient rights focus, i.e., decisional capacity, consistent with the law. Future experience will show whether and how this change will be integrated into clinical practice. In this process, institutional ethics policies may—in addition to the law, national guidelines, or medical standards—be helpful in addressing conflicting duties at the bedside. The article offers an interdisciplinary theoretical reflection with practical illustration.

## 1. Introduction

In the last decades, Switzerland has gained the reputation of being unique with regard to the practice and tolerance of assisted suicide (AS)—unique in comparison to other liberal states, e.g., the Benelux states permitting euthanasia and physician-assisted suicide. Only Switzerland permits lay right-to-die societies (RTDS) to organize AS with physicians playing a marginal or formal role, attesting decisional capacity and prescribing a lethal dose of Natrium-Pentobarbital to the patient, whereas the personal accompaniment into the dying process is overtaken by a nonclinical member of the RTDS [1,2,3]. Swiss RTDS consider themselves not as medical or professional associations, but as lay volunteers. Nevertheless, Switzerland has been a country with a strong commitment to suicide prevention and displays highly developed and institutionalized health care facilities. These coexistent perspectives on the topic of suicide create a clash of values and this clash of values contributes to emotional discussions, both in public as well as at the bedside. 

Thus, two ethics polices for the University Hospital Basel (USB) were initiated. Addressing the evident tension, they offer the possibility that health care professionals can find a consistent and integrative way of responding to challenges in both directions: respect and tolerance regarding a patient’s wish for AS on the one hand, and the determination to offer help and prevent harm by practicing suicide prevention. In a broader perspective, ethics policies are considered as options of clinical ethics support including also ethics consultation, staff-related educational activities, etc. [4,5,6]; e.g., national medical ethical guidelines, which also count as ethics support.

Against this background, the development of **Policy 1 on Assisted Suicide** was started. Moreover, a motion at the local Parliament called after the initiator, the politician Luca Urgese, contributed to the need for clarification. He claimed a general patient right to commit AS in any publicly funded hospital of the federal state of Basel City. Finally, this motion was rejected [7]. The outlook to potentially becoming involved in organizing AS on the ward stimulated concern and even resistance among some members of clinical staff, while others felt that this “patient right” could and should not be denied. This controversy was taken up by the Advisory Ethics Board (EAB) of the USB with the decision to produce an ethics policy for in-house orientation. In sum, the ethics policy offers support and access to ethics consultation in situations or cases of uncertainty, and defines the room for understanding, empathy, and tolerance regarding a patient considering AS, but also defines the limitations: AS must not be performed in the USB; suicide prevention continues to be explicitly and fully embraced in the clinical obligations. However, while dealing with a patient’s request for AS—in the framework of a “rational decision”—is a rarely occurring challenge in acute patient care, the necessity of caring for patients after a suicide attempt or responding to suicidal symptoms such as loss of hope in severely ill patients appropriately represents a classical and quite frequent task of clinical care including nursing. 

As a result, **Policy 2 on Suicide Prevention** was initiated by a group of nurses from the (somatic) USB in collaboration with the Psychiatric University Hospitals Basel (UPK) with the support of the Nursing Directors. All health care professionals may face patients with suicidal ideation, but nurses are in a prime position to identify patients who may be at risk. This policy aims to assist nurses and other health care professionals working in acute care to provide evidence-based care to adults at risk for suicidal ideation and behavior as well as patients after a suicide attempt. It also addresses strategies to promote wellbeing for the patient, his or her family, and the nurse.

In this theoretical article, we will describe the twofold requirements for clinical and nursing staff: corresponding with policy 2, to build up competencies of responding appropriately to suicidal patients, i.e., taking necessary decisions and actions, and, corresponding with policy 1, to develop the capability to communicate in a respectful and empathic manner with patients requesting AS, acknowledging their personality and the liberal societal context of Switzerland, even if (morally) disapproving the choice. In the communication with a patient raising the topic of suicide, one major difficulty lies in the—preliminary—classification of the patient as “suicidal” and, thus, in need of protection and treatment or as a person with the decisional capacity and the right to choose AS. The approaches of policy development will be described as well as the respective policy content. Their practical relevance will be illustrated by authentic clinical vignettes highlighting the need of health care professionals for guidance when facing issues of suicide, here: by an institutional policy. Moreover, the vignettes shed light on which practical and ethical questions can be answered with the help of the policy and which bedside challenges are left to individual reflection and coping. Vignettes A and B come from the practice of Ethics Consultation in the Basel University Hospitals [4] and were written by the first author (S.R.T.), while vignettes C and D represent experiences of nursing practice and were contributed by the last author (I.A.F.). In the discussion, we will contextualize the situation in light of the legal framework and the (recently revised) Swiss Academy of Medical Sciences (SAMW) guideline with the aim of offering an ethical reflection of the policies’ significance and the societal situation in which they are currently embedded.

### Vignette A—Dealing with a Suicidal Patient’s Reported “Wish to Die”

An acute ethics consultation (EC) is requested urgently from the emergency department. The physician in charge and his team are concerned about the threat of a patient’s husband: He had called the emergency to catch his unconscious wife after taking an overdose of sleeping pills. When arriving on the ward, he forbids undertaking life-prolonging measures, claiming that his wife, a member of an RTDS, wanted to die. Being a lawyer, he threatens starting a lawsuit claiming that he is his wife’s legal substitute decision maker. Outcome of EC: The legitimacy of making such a decision in the husband’s role is challenged. Formally, his role seems to be consistent with the law, but he fails to provide convincing reasons why his wife should not receive life-supporting measures. It is argued that the law requires the substitute decision maker to consider the presumed patient’s wishes, and, if not known, represent the patient’s best interest. As the middle-aged patient has a psychiatric history of depression, it is concluded that an obligation exists to explore the patient’s authentic motivation after regaining decisional capacity and offering antidepressant treatment before eventually taking suicidal wishes as valid. (Due to the patient’s rapid stabilization, no life-prolonging measures had to be taken).

At the time, the position of (proxy) substitute decision makers had recently been strengthened by law and no institutional policy had been established yet. Vignette A mirrors the conflict that may be experienced at the bedside between the duty to respect a patient’s (substitute decision maker’s) wish and the duty to protect the patient from self-harm. The need for guidance was expressed in the request for an EC. Recommendations such as policy 1 on assisted suicide as well as policy 2 on suicide prevention would have been helpful as well. 

## 2. Development of Two Institutional Policies: (1) On Assisted Suicide and (2) On Suicide Prevention

### 2.1. Policy 1 on Assisted Suicide 

In 2013, the interdisciplinary Ethics Advisory Board (EAB) decided to develop an ethics policy on AS to help health care professionals handling patient AS requests. Not only did the ongoing debate on AS trigger ethical questions on the subject, but also practical challenges at the bedside motivated the EAB to take the initiative. Moreover, the possibility emerged that as a public hospital or a health care professional, one might be driven into becoming practically involved in AS intra muros (by political decision). The existing ethics consultation service should not suggest that issues of AS were to be handled on a case-wise basis only. Rather, some more general guidance for a joint in-house attitude was required given the controversial and less-than-clear situation regarding AS and its regulation in the country [1,2,3,8]. Thus, the EAB proactively prepared a written institutional policy. Starting with a first draft (provided by SRT) referring to the legal situation, available documents, and clinical experience, several draft versions were submitted to discussion and review by the EAB including representatives of the hospital’s legal and compliance office as well as chaplaincy. Members of RTDS were not involved in the process as no USB external experts had been invited. The final version, dated 16 March 2015, was put on the agenda of the USB Board of Directors for discussion. Approval was obtained 18 May 2015. The policy was posted on the website for internal and external access. 

The policy is based on a summary of the current legal and guideline framework regulating AS in Switzerland. It further refers to the existing right-to-die societies (RTDS) offering organized AS acknowledging the leeway of physicians getting involved with RTDS and AS outside the USB. As a core element, the expectations are articulated that the USB-employed health care professionals have to fulfill towards AS requests, i.e., the dos and don’ts. 

In the process of review and revision, controversial and divergent views from within the EAB and the USB in general were acknowledged. Similarly to society at large, different views were brought forward trying on the one hand to strengthen patient autonomy and support respecting patient choices for a self-determined way of dying. On the other hand, a great effort was taken to confirm the hospital and its staff to be safe from intrusion by RTDS or irritation about AS practice in patient care. Given the wide span of values under this umbrella, the resulting policy does not take the side of one single party. Rather, it articulates the free space that a health care professional has in responding to a patient’s AS request, as well as its limitations. Moreover, it formulates basic procedural aspects, especially of open communication: health care professionals are supposed to respond with empathy to the patients’ basic needs. Last but not least, the policy offers the EAB’s practical support and consultation to those involved if needed.

### 2.2. Policy 2 on Caring for Patients Who Express Suicidal Thoughts

In 2015, the USB Board of Directors mandated an interdisciplinary working group experienced in practice, research, and ethics from both the USB as well as the Psychiatric University Hospitals Basel (UPK) to develop and implement a policy to support nurses in caring for patients at risk for suicidal ideation and behavior. Based on the ethics guideline of the Swiss Association of Nurses and in correspondence with the International Council of Nurses (ICN) [9] code of ethics, nurses have the responsibility to promote health, to prevent illness, to restore health, and to alleviate suffering [10]. The following questions were guiding the project: Which risk factors can indicate suicide? By which screening tool can suicidal ideation and behavior be assessed? What are effective nursing interventions to prevent suicide or suicidal behavior? Which strategies can be recommended for nurses to cope effectively with emotional distress? Subsequently, a literature search for clinical guidelines and recent articles (in German language) was conducted. The working group developed the policy and submitted a draft to nurses and other clinical staff members as well as the EAB for review. Their feedback was acknowledged in the revision. Approval was obtained from the Nursing Management Conference (7 June 2017) and the policy was published on the website (7 September 2017) accessible from inside as well as from outside.

For the development of the two policies, the legal, ethical, and health care context, internal evidence, such as interdisciplinary professional knowledge, and clinical experience, as well as generally available evidence were acknowledged [11]. Clearly, both policies are different from medical guidelines focusing mainly on serving the Basel University Hospitals’ and their staff’s needs for guidance. While policy 1 has been a project of the EAB, the nursing profession played a core role in policy 2, often being the first at the bedside to hear about wishes to die, recognizing suicidal symptoms, or being the primary caretakers for patients after a suicide attempt. Patient perspectives, experiences, and preferences were considered by listening to and reflecting on their stories. 

### 2.3. Legal Context of Suicide Prevention and Assisted Suicide

#### 2.3.1. Suicide Prevention

So far, a national program for suicide prevention is yet to be established in Switzerland. However, since 2014, efforts have been made to develop an action plan for suicide prevention [12]. A survey performed earlier showed that suicide prevention measures in Switzerland were generally sparse; for the elderly they were even lacking [13].

#### 2.3.2. Assisted Suicide

The legal basis for AS is finally regulated by Art. 115 StGB (criminal code) [14]: immunity from punishment for assisting in the suicide of a patient requires (1) the person being assisted in suicide to have decisional capacity and (2) that suicide is committed by choice and inheriting authority of action, i.e., control (“Tatherrschaft”)—the latter requiring that the actions leading to suicide and death be performed fully by the patient. Suicide assisted by legal persons or RTDS must be without interest motives (the law says “selbstsüchtige Motive”/“selfish motivation”). In addition to this legal norm, court decisions and recommendations concretize legal provisions. According to federal court decision [15], Natrium-Pentobarbital (NaP) can be prescribed in lethal dose by MDs (based on Art. 24 and 26 HMG (Heilmittelgesetz) and Art. 9 and 10 Abs. 1 BetmG (Betäubungsmittelgesetz), respectively). Medical justification (namely by diagnosis, indication, patient-informed consent including conversation, attested decisional capacity, discussion of treatment options) of AS is to be guaranteed by prescription requirement. Furthermore, this federal court decision points out that the prescription of a lethal dose of NaP is underlying the medical association’s professional code of conduct, especially the rules of the SAMW. 

The SAMW medical ethical guideline for end-of-life care issued in 2013 [16] was submitted to revision in 2017. Before completion of the revision, the existing guideline (of 2013) required that any physician deciding to assist suicide is responsible to ensure the following criteria:

– The underlying disease implies that the end of life is near.
– Alternative supportive measures were discussed and realized, if desired.
– The patient has decisional capacity, the decision for suicide is well considered and long lasting, and the patient must be aware of the consequences and act uninfluenced. This condition should be confirmed by a third person (not necessarily by an MD).

The title of the revised guideline (published: 6 June 2018) [17] was changed from “End of life care” into “Management of dying and death”. The requirements made in the revised SAMW guideline read as follows ([17], quoted from p. 23):

If an autonomous desire for suicide persists in a patient who has been carefully informed and assessed, a physician may—on the basis of a decision for which he or she is personally responsible—perform assisted suicide, having verified that the following five requirements are met; it must be additionally confirmed by an independent third party (who need not be a physician) that the first two requirements are met:

1. The patient has capacity in relation to assisted suicide. It must be documented that *in*capacity has been carefully excluded by the physician. If a mental disorder, dementia or another condition frequently associated with lack of capacity is present, capacity must have been assessed by an appropriate specialist.
2. The patient’s desire is well-considered, not due to external pressure and enduring. If there is evidence of a problematic relationship of dependency, careful consideration must have been given to its possible influence on the desire for suicide.
3. The symptoms of disease and/or functional impairments are a source of intolerable suffering for the patient.
4. Medically indicated treatment options and other types of assistance and support have been sought and have proved ineffective or are rejected as unacceptable by the patient, who has capacity in this regard.
5. The patient’s desire not to continue living in this situation of intolerable suffering is comprehensible for the physician on the basis of the previous history and repeated discussions, and the physician finds it justifiable to perform assisted suicide in this particular case.

In contrast to the draft revision version, the criteria listed in the final guideline start with (1) the patient’s decisional capacity instead of the previously first point, “The underlying disease implies that the end of life is near.” Accordingly, “intolerable suffering” becomes criterion (3), replacing “end of life”.

Based on unclear requirements for AS and the fact that RTDS justify their legitimation upon Art. 115 StGB [18], revision of Art. 115 StGB was considered to achieve full national legislation on authorization and regulation of RTDS. However, this consideration was declined by the federal council. Furthermore, federal lawmakers renounced national supervision of RTDS by supervisory law and adherence to certain mandatory accuracy principles as this approach appeared disproportional [19]. Moreover, it was criticized that a legal framework would have suggested institutionalization and legitimization of RTDS. 

## 3. The Two Policies and Their Practical Illustration 

Policy 1 aims at offering guidance and clarification on how health care professionals are supposed to respond to patient requests regarding AS (Box 1) [20].

Box 1 **Policy 1.**

**Guidelines for health professionals: Handling of patients’ request for assisted suicide**

1. **Respect and empathy**

Patients’ autonomy is a high value; personal decisions are respected at the University Hospital Basel. This also applies for difficult decisions at the bedside, which are perceived as ethically controversial. Responding in an ethically correct manner and with empathy upon a patient’s request for assisted suicide poses a special challenge to health care professionals.

2. **Contact and offer for conversation**

A patient’s wish to discuss assisted suicide is to be accepted openly and without reservations or judgment by health care professionals. If necessary, e.g., in case of uncertainty or the feeling of over demand, more experienced colleagues may be involved to conduct the conversation. This conversation preferably takes place without delay.

3. **Access**

Patients may receive visitors from right-to-die organizations; the purpose of the visit has to be personal and individual.

4. **Qualified ethical support**

The Ethics Advisory Board can be consulted upon request. Consultations are confidential. Requests and ethics consultations are being documented. Also, the consultation of interdisciplinary specialists (e.g., palliative care, pastoral care, or psychological support) is possible. The Ethics Advisory Board consists of experienced and qualified ethics consultants who provide expertise as well as respectful and supporting consultations.

5. **Scope of support at the University Hospital Basel**

Assisted suicide is not performed at the University Hospital Basel. Yet, with respect for a patient’s decision, we offer discussion and consultation about treatment options, as well as medical clarification (e.g., prognosis or a patient’s decision-making capacity). Based upon expertise in palliative care and ethical analysis, ethics consultation can as well deal with the issue of dying with dignity.

Generally, health care professionals at the University Hospital Basel are not obliged to provide information on assisted suicide. However, giving support by providing information can be appropriate in case the requesting patient is unable to gain information autonomously. Practical support (e.g., logistics) is possible in case a patient decides to commit assisted suicide with the assistance of a right-to-die organization elsewhere.

6. **Legal framework**

With regard to individual liberty and freedom of choice, suicide committed by a person with sufficient capacity of discernment is not liable to prosecution. Generally, seduction to (inducing the decision to suicide with intent) and assistance (any form of contribution with intent) in suicide generally are not liable to prosecution, whereas seduction to and assistance in suicide with selfish motives are. Under the assumption that organisations offering suicide assistance generally have no interest motives, the assistance in suicide is legal. Immunity from punishment for assisting a mentally ill person to commit suicide is legally disputed, as any suicidal person must be aware of the consequences and act uninfluenced. Active euthanasia is always punishable by law, even if performed upon serious and urging request and by honourable motives, as no legal consent exists.

Criminal law and professional ethical guidelines imply no obligation to contribute to assisted suicide. As mirrored by the medical association’s professional code of conduct, physicians are not obliged to perform any medical treatment contrary to their conscience. Whether or not the right to self-determination at one’s end of life entitles the person to the right to be assisted in committing suicide by the state (or public hospitals), be it by appropriate facilities or staff is not defined.

7. **Prevention of suicide**

This guideline does not challenge the importance of prevention of suicide and crisis intervention work for persons in need, e.g., depressive patients.

8. **Outlook**

Based upon this guideline, experiences with requests and consultations will be analyzed to further develop the process and adapt to the needs of persons involved.

Ethics Advisory Board, University Hospital Basel, March 2015

**References: see German version**

The following vignette illustrates ethical and practical challenges occurring with requests for AS. 

### 3.1. Vignette B—Ways of Getting Involved in AS

A patient in his late fifties suffering from severe chronic pain is transferred to outpatient EC. (Outpatient ethics consultation here is an exceptional service, offered under particular circumstances). He has triggered perplexity in several interdisciplinary clinical teams of various hospitals. While his original request was to obtain an attestation of decisional capacity required by a right-to-die society before they would give him assistance to commit suicide, he confirms, on closer exploration of the ethics consultant, that he might prefer to live if his permanent pain were eased. Outcomes of EC: As a consequence, the ethics consultant engages in finding another hospital/specialty willing to open his record again and to address his pain. After a lengthy sequence of diagnostics and treatment attempts (and only partly successful pain control), the patient receives the desired attestation of decisional capacity from an external palliative care unit, i.e., the prerequisite for obtaining a lethal dose of Natrium-Pentobarbital and accompaniment into death by the RTDS. As a further result of the EC, his primary care and home nursing situation can be modestly improved. Catamnesis proves that he continues to live after 4 years saying that he prefers to not commit AS for as long as possible, thus preventing burdening his next of kin. In accordance with policy 1, the patient’s wish to find support for an AS was respected and handled with empathy. However, practical in-house assistance to commit suicide was not offered. Following the prerequisites of the SAMW guideline, available treatment options were evaluated and tried as well as an attestation on decisional capacity provided by an external service. Any attempt to treat the patient sensu strictu as a suicidal person met the patient’s resistance who declined all kinds of psychiatric or related services.

### 3.2. Policy 2 on Suicide Prevention

This policy aims at giving advice and recommending strategies for nurses to respond appropriately to patients with suicidal ideation (Box 2) [21].

Box 2 **Policy 2.**

**Guideline for nurses in clinical practice: Caring for patients with suicidal ideation**

1. **Scope**

To set out a framework for interacting with patients who express thoughts of suicide or exhibit self-harming behavior and to describe the associated nursing interventions.

2. **Aims**

Recognizing behavior of patients with suicidal, self-harming intent and ensuring professional nursing care.

3. **Background**

Health care professionals meet patients who are hospitalized as a result of a suicide attempt or are suspected of having suicidal behavior. Nurses play an important role in recognizing suicidal intent as well as in preventing self-harming behavior. Caring for this group of patients can be challenging and confronting nurses with their ethical considerations regarding care, autonomy, and respect for individual decisions made by patients.

4. **Definitions and risk factors**

The term suicidality is used to describe the tendency towards suicide. In a more general sense, suicidality is a collective term characterizing an individual’s pattern of thoughts and behavior that may be auto-destructive in nature and that, either directly or indirectly, accede to the idea of the individual’s own death. Risk factors are: history of suicide attempts; expressing suicidal thoughts; mental disorders such as depression, addiction, post-traumatic stress disorders; somatic symptom disorders with minimal chance of recovery and/or high risk of fatal outcome; adverse life events such as unexpected loss of a loved one, job loss, being diagnosed with a life-altering disease; feelings of desperation, feeling overwhelmed, hopelessness, loneliness, and/or feeling worthless; older men have the highest risk of suicide, young women the highest risk of attempted suicide.

5. **Nursing interventions**

Caring for patients with suicidal ideation requires a sound level of competence and an interdisciplinary and inter-professional teamwork. In reference to the aim, this guideline limits itself to describing nursing interventions.

– Assessing patient’s situation and reporting measures to the ward physician and the psychiatric consultant for clinical reasoning. The assessment criteria of the *Nurses’ Global Assessment of Suicide Risk* can help the nurses collect information in a concise manner and be added to the psychiatric consultation.
– Interacting with patients and agreeing on contact at regular intervals and reporting if suicidal thoughts become more pronounced.
– Informing, integrating, or involving relatives or friends in accordance with the patient.
– Continuing information about actions and adhering to the agreements.
– Continuity of care has to be guaranteed whenever possible.
– Binding agreements about mobility and precautionary safety measures.
– Selecting the patient’s room depending on the symptoms exhibited and suicide risk level.
– Regular contact with the psychiatrist to follow up.
– Documentation of the care process.

6. **Coping with burdensome situations**

Caring for patients who express suicidal thoughts can be very challenging. Opportunities to discuss and structurally reflect stressful situations and experiences in the interdisciplinary and inter-professional team are vital.

**References: see German version**

Many nurses in acute care settings have experience with patients who express thoughts of suicide or exhibit self-harming behavior in the course of their illness trajectory, and there are also patients admitted to the hospital after an attempted suicide. A need for orientation by guiding principles in caring for these vulnerable patients has been clearly articulated. The first group is illustrated by Christine, a nurse on the neuro-surgical unit.

### 3.3. Vignette C—Confronted with Suicidal Thoughts

Christine reported a situation with a 64-year-old male patient, recently retired, who was admitted to the neuro-surgical ward. After diagnostic procedures, a brain tumor (Glioblastoma Grade IV) was diagnosed. The patient showed a wide range of emotions, but mainly anger at the tumor and the related life perspective. This situation made him express suicidal thoughts, however, without distinct ideas to terminate his life. Communication with the patient was experienced as straight, but always with underlying aggression because of hopelessness and loss of control. However, the interaction with his relatives was challenging since they were in a state of neglect. Psychiatric consultation and medication relieved the tension and with support of social care the patient could be transferred to a specialized institution for recuperation as soon as there was no further need for acute care. 

In retrospect, Christine reflected this situation using the recently enacted policy. She concluded that to know how to approach achieving an agreement with the patient regarding his suicidal thoughts would have been helpful. Besides that, she felt well supported by the unit’s physicians and the psychiatrist. 

Some of the challenges of a health care team triggered by patients after an attempted suicide are expressed in the following example. 

### 3.4. Vignette D—Dealing with a Patient’s Withdrawal

A young female patient was admitted to the hospital after a suicide attempt by jumping out of a window. She had been in psychiatric treatment because of a depression and severe mobbing at her workplace. The injuries incurred were severe with multiple fractures causing an incomplete paraplegia. Her situation presented many challenges since she needed intensive caring and required two to three nurses at night for positioning, but refused to accept certain nurses. According to the nurses, this patient avoided contact and preferred to be in her shaded room although she was much in need in terms of pain management and wound care.

The nursing team’s reflection was that the challenges were manifold. Thus, the unit-based Clinical Nurse Specialist (CNS) called a case conference inviting the unit’s physicians, the psychiatrist, the pastoral care giver, the primary nurse, as well as the unit manager. Reflecting on the patient’s situation from the different perspectives led to new insights and varied interventions. While this case conference was important and helpful, the CNS was thoughtful by reviewing the new policy. A closer collaboration with the psychiatrist as well as negotiating a schedule for regular patient contact and building consensus about nursing interventions would have been even more helpful if they had been in place from the very start. 

## 4. Discussion

It has been shown in the paper that the coexistence of tolerance for *assisted suicide* (AS) in Swiss society and the necessity to provide *suicide prevention* creates a tension between the underlying values and may also trigger ethical uncertainty among health care professionals: When does a patient deserve respect, or even support, for the wish to die by terminating his or her life, and when is it the primary duty to hinder a person from committing a suicidal act—and how should this be done? Questions such as these reveal the basically controversial and value-laden nature of suicide that does not allow for a simple answer. Obviously, Swiss legislation has not offered a clear path either. National guidelines might play an important role in clarification, but the SAMW guideline mirrors the normative tensions. With regard to suicide prevention, only few legal criteria relevant for the health system are available to determine in which situations health professionals are obliged to prevent suicide. Furthermore, it also needs clarification to what extent the health system itself has the function and competence to offer and develop forms of suicide prevention. 

The development of the two institutional policies presented here is quite different from the methodology of medical guideline development [22,23]. As “ethics policies” [24], they should be evaluated according to their objectives; their primary value, we suggest, lies in their contribution to clarification. Moreover, ethics policies count as one—sustainable—form of clinical ethics support and are supposed to serve those involved by offering help for the oftentimes demanding reflection of the normative dimension of health care [25]. Both policies do reach their goals, but to different extents: policy 1 on assisted suicide formulates a consistent in-house attitude preventing ethical conflict, but cannot eliminate the persisting societal controversy on AS. Policy 2 on suicide prevention successfully provides a frame of reference and practical advice most helpful for nurses at the bedside. Challenges remain for all clinical staff wishing to take their patients’ wishes seriously: the distinction is by no means easy to make between a patient who depends on being rescued and protected from self-harm by suicidal acts on the one side versus a patient on the other side seeking empathy and support for a “well-considered” (SAMW) decision to die through AS. There is still belief that the wish to die must always be “sick”, i.e., a symptom of “psychopathology” and, thus, always an indication for treatment, even by coercive measures against the patient’s wishes. While this concept may be clinically valid in individual situations, it cannot be claimed to be generally valid and applicable without subscribing to medical paternalism and falling behind patient rights of self-determination as guaranteed by Swiss civil law. For the difficult distinction to be made at the bedside—duty to respect versus duty to protect—health care professionals may consult the SAMW guideline. In the established revision, the previously leading clinical criterion allowing for AS in a patient whose “end of life is near” has been replaced by “intolerable suffering”, opening up a much larger group of patients; this corresponds to data published in 2009 emphasizing the significance of pain and unbearable suffering [3]. Additional to this liberalization, the decisional capacity (attestation) has been formulated more precisely. Capacity, newly mentioned first in the revision, is the only criterion of this list that is also incorporated in the existing legal framework (Art. 115, Swiss Criminal Code). 

While the development of policy 1 has absorbed the controversial character of the topic of AS embedded in society, the Board of Directors’ decision to enact it unanimously acknowledged the policy’s consistency and integrative function for the institution. Little is known about situations where the USB staff is put to the test of handling AS requests appropriately—outside ethics consultations—and how helpful the policy actually has proven at the bedside. In a political or institutional sense, policy 1 may be called useful as it appears to have put the experienced uncertainty—“what shall I as a staff member do?”—to an end, even though pluralistic views on AS are persisting inside and outside the USB. Experiences did inform the writing of the policy as well as reports from other Swiss institutions which did or did not formulate policies on AS, restrictive or liberal. Lausanne University Hospital (CHUV) and Geneva University Hospital (HUG) were the first Swiss University Hospitals to (voluntarily) enact policies on admitting RTDS to organize AS within the hospital as early as 2006—examples that were not followed by other University Hospitals in the country. Early clinical case reports from the CHUV suggested that in-hospital AS turned out to be a greater challenge than had been expected [26]. Other examples for liberal institutional policies regard nursing homes; here, the canton Zürich was pioneer [2,27,28]. Geneva just adopted a law in May 2018 by acceptation by the Grand Conseil going into a similar direction. One core reason presented as justification for such liberal decisions focuses on fairness: patients who do not have households anymore and live in the nursing home shall not be put at disadvantage by denying them the right to carry out AS in the facility. A similar reason is applied in the case of CHUV: patients who are not transportable shall have the same right to perform assisted suicide in hospital [26]. Waadt’s decision to enact the obligation that publicly funded health institutions in the whole canton have to accept AS under certain prerequisites and that RTDS have to get access to organize AS (Art. 27 d LSP, health law, canton Waadt) has remained an exception. Thus, the overall picture in Switzerland is characterized by heterogeneity allowing citizens in the ideal case to choose, e.g., a nursing home that is in accordance with their values. However, it may be assumed that even in countries with a less ambiguous legal framework of AS, societal controversy may continue [1,2]. “German law does not criminalize suicide or persons helping in a suicide, but in November 2015, Germany forbid assistance in facilitating suicide in a commercial or business-like form, as available in Switzerland” ([29], p. 80); however, the debate is continuing in Germany as well, now focusing on questions such as what counts as “commercial or business-like form”. For the special Swiss situation, it appears desirable that health care institutions should take the initiative to transparently clarify their own situation and attitude so that patients, clients, and even health care professionals have the chance to decide whether or not they can adopt and live up to the respective policy. This freedom of choice evidently does not exist in most Swiss cantons, except Waadt and Geneva. As both convictions are strongly rooted in Swiss society—the duty to respect AS and the duty to prevent suicide—it might contribute to conflict resolution if institutions in one area reflect different sets of values: some more liberal and others more restrictive. Such an approach towards societal adjustment comes, of course, by the price of lacking (national) consistency. 

Regarding clinical practice, the first two vignettes, A and B, highlight the need for guidance felt by health care professionals when facing the topic of AS with their patients. Vignette A highlights that a patient’s presumed wish to commit AS (reported by her husband) must not be “believed”, but has to be explored thoroughly and authentically according to established criteria such as decisional capacity, intolerable suffering, etc. (SAMW). In vignette B, the professional duties were more ambiguous or rather twofold: the patient’s wish for AS was explored in light of the criteria: severe pain, unresponsive to treatment (see above: SAMW revision), has been corroborated as well as decisional capacity and procedural requirements. Thus, AS becomes accessible. However, the patient has been encouraged to take AS as his very last resort and, adopting this view, still continues to live. Vignettes C and D focus on the need for help in fulfilling the duty to protect patients from self-harm and suicidal acts, thus, preventing nurses’ uncertainty and distress. On the whole, we consider it necessary and possible to address both obligations—the duty to respect and the duty to protect—in institutional policies from a practical and ethical perspective, as has been done in the USB. 

With Policy 1 becoming visible in the hospital and the topic of AS being in the media oftentimes, policy 2 on suicide prevention seemed to be the missing answer to manifold questions at the bedside. It helped to complement the picture and acknowledge the two sides of the conflict. As vignettes C and D illustrate, the challenges, especially for nurses, to responding appropriately to patients who express suicidal thoughts or after a suicide attempt remain considerable. Caring for these patients demands taking patients’ statements seriously, being sensitive to risk factors, and planning and conducting care according to the agreement in the interdisciplinary team and with the patient [30]. In the course of developing the policy, feedback from nursing teams and CNSs helped to refine and shape supportive interventions. Using policy 2 for orientation allows for a more structured assessment supporting clinical reasoning in the interdisciplinary team and planning interventions tailored to the patient’s needs. In this respect, the inclusion of relatives can be of paramount importance, but needs to be permitted by the individual patient. Communication with patients in such a vulnerable state requires a certain level of proficiency and reflection. Case conferences with different professionals or Ethics Consultations [4,5,6] have been in place and prove to be important for exploring situations as described in all vignettes and for determining an appropriate care plan. Providing formal and emotional support for health care professionals to enable them to provide good care is an institutional responsibility [31]. In this respect, feedback has shown that before policy 2 was established, such support was lacking and called for further action. Education and clinical supervision, facilitated by an advanced practice mental nurse at the bedside, could also help nurses to become more competent and confident in caring for suicidal patients. Both policies’ outreach is, to our knowledge, limited to the USB. However, in the Swiss context, this focus does not have to be a disadvantage. Rather, such institutional guidelines have merits: the institution has control over the enactment, the evaluation of its consequences, any revisions, and conclusions. 

We have shown that the clinical and ethical challenges related to assisted suicide and suicide prevention cannot be understood without acknowledging the unique legal and societal Swiss context. One prominent function of legislation in society is seen in the elimination of normative conflict and vagueness. To which extent has this goal of clarification been realized for AS and suicide prevention in the Swiss context? While according to medical deontology providing suicide prevention is clearly a part of the professional obligations, in AS fundamental questions are still open, e.g., “which indications for AS are accepted?” and “can any physician prescribe NaP?” ([32], p. 76). The same author also asks “which level of decisional capacity is required?”, although—legally speaking—capacity is either (1) presumed or (2) has to be denied according to certain criteria, and the author is a lawyer; however, it has become established clinical practice to explore, especially in demented patients, whether there is understanding—despite attested incapacity—of the consequences of a specific decision or action, e.g., forgoing life-sustaining treatment (implying that there may be a “level” of capacity). In the case of AS, this concept may lead to confusion as patient capacity is required in full for accepting AS, admitting no “levels” or grey zones. Even though the federal court considers the legal norms for prescription of NaP as suitable to provide a certain amount of control through the involved medical professional and the according obligations, the above-mentioned questions yet remain unanswered. The steady involvement of physicians in decision-making and AS preparation processes stands in contrast to the dominance of nonclinical members of the RTDS, actually and practically assisting suicide by administering NaP and yields reason for discussion [8]. In international comparison, it is astonishing that the assumed wish of Swiss society to tolerating assisted suicide is not narrowed down to “physician-assisted suicide” including public control, but that by concept of the RTDS—and tolerated by the state—health professionals are only marginally involved in the latter process. Legislators decided to neither specify Art. 115 StGB nor issue national regulations for RTDS. The rationale for this decision was to avoid providing governmental legitimation for RTDS. This corresponds with the SAMW guideline’s final revision: while the preliminary revision version had referred to RTDS by saying “suicide can be assisted by a single MD or in collaboration with an RTDS given the following prerequisites…”, the final revision does not point at RTDS for potential transferal. By dropping the reference to RTDS and transferal, the SAMW, too, avoids their (implicit) legitimation. It is put to discussion whether the “unique” Swiss situation may shed some light on the challenges faced even in other countries where the topic of AS becomes more popular. 

## 5. Conclusions

1. Developing an institutional ethics policy may be a useful approach to clarify the appropriate way of responding to patients’ requests for assisted suicide.
2. Suicide prevention is a clear duty of health care professionals; however, professional handling of suicidality is full of practical challenges that can be eased by a policy.
3. The increasing presence of assisted suicide increases the need for advice about making important distinctions and following rational criteria such as patient rights and capacity as well as professional obligations.
4. Institutions facing related difficulties are encouraged to share experiences and documents about developing policies tailored to their context.
5. As societal controversy cannot be silenced by policies, the clarification of expected in-house attitude and practice becomes even more important to prevent ethical uncertainty and conflict.
